# The ACTH test fails to diagnose adrenal insufficiency and augments cytokine production in sepsis

**DOI:** 10.1172/jci.insight.187487

**Published:** 2025-03-06

**Authors:** Dan Hao, Qian Wang, Misa Ito, Jianyao Xue, Ling Guo, Bin Huang, Chieko Mineo, Philip W. Shaul, Xiang-An Li

**Affiliations:** 1 Department of Pharmacology and Nutritional Sciences,; 2 Saha Cardiovascular Research Center, and; 3 Division of Cancer Biostatistics, Department of Internal Medicine, University of Kentucky College of Medicine, Lexington, Kentucky, USA.; 4 Department of Pediatrics, University of Texas Southwestern Medical Center, Dallas, Texas, USA.; 5 Lexington VA Healthcare System, Lexington, Kentucky, USA.; 6 Department of Physiology, University of Kentucky College of Medicine, Lexington, Kentucky, USA.

**Keywords:** Infectious disease, Inflammation, Cellular immune response, Innate immunity

## Abstract

The adrenocorticotropic hormone (ACTH) test diagnoses relative adrenal insufficiency (RAI) or critical illness–related corticosteroid insufficiency (CIRCI). Initially, guidelines recommended corticosteroid/glucocorticoid (GC) therapy for septic patients with RAI, but later trials did not show a survival benefit, leading to updated guidelines that abandon targeting RAI or CIRCI. Recent studies with an RAI mouse model showed a clear survival benefit from GC therapy in mice with RAI, suggesting that inconclusive GC clinical trials might be due to issues with the ACTH test rather than targeting RAI. To investigate, we performed the ACTH test in septic mice. Interestingly, the ACTH test identified most mice as having adrenal insufficiency in early and middle stages of sepsis, even those with a normal adrenal stress response. Surprisingly, the ACTH test increased inflammatory cytokines to lethal levels, moderately increasing mortality in septic mice. This study revealed significant flaws in the ACTH test for diagnosing RAI/CIRCI. It not only fails to correctly identify these conditions, leading to misguided use of GCs, but also induces a lethal inflammatory response in sepsis. These findings suggest that inconclusive GC therapy trials may be due to the problematic nature of the ACTH test rather than ineffectiveness of targeting RAI/CIRCI.

## Abstract 2

This study reveals flaws in ACTH test for diagnosing adrenal insufficiency in sepsis, underscores a need for a new diagnosis and reevaluation of glucocorticoid therapy.

## Introduction

Sepsis is a major health problem, claiming 11 million lives each year (1). Adrenal insufficiency is common in sepsis (2). It is classified into 3 types: (a) absolute adrenal insufficiency (low plasma cortisol levels at <100 ng/mL, ref. 3); (b) relative adrenal insufficiency (RAI), defined by insufficient production of cortisol relative to organ demand and diagnosed with a change in cortisol (Δcortisol) of less than 90 ng/mL after an adrenocorticotropic hormone (ACTH) test (4); and (c) glucocorticoid (GC) resistance (impaired cellular GC signaling) (5). RAI is the most common in septic patients, affecting 25% to 60% (6). In 2008, a task force introduced the term “critical illness–related corticosteroid insufficiency (CIRCI)” to replace “RAI” (3). CIRCI is defined by inadequate cellular corticosteroid activity for the severity of the patient’s critical illness, diagnosed when a seriously ill patient has very low cortisol levels (<100 ng/mL or a Δcortisol < 90 ng/mL upon ACTH stimulation test). Despite some disagreement, the ACTH test is commonly used to diagnose CIRCI (7).

Annane et al. used the ACTH test to identify septic patients with RAI and found that those treated with steroids had a lower death rate after 28 days. This became known as the French trial (8). The 2004 Survival Sepsis Campaign Guidelines adopted the ACTH test to identify RAI and suggested GC therapy for patients with RAI (9). However, the CORTICUS trial did not confirm the benefit of GC therapy in a less severe group of septic patients with RAI (10). As a result, the later guidelines did not recommend the diagnosis of RAI with the ACTH test and suggested GC therapy for patients based on shock status rather than RAI (11–13). Despite weak recommendation, about 50% of septic shock patients have received GC therapy (14–17). The recent ADRENAL trial, which included 3,800 septic shock patients, found no survival benefits from GC therapy (18). Notably, the trial administered GCs to patients without performing the ACTH test to distinguish their RAI/CIRCI status. In contrast, the APROCCHSS trial found an improved survival rate in septic patients treated with GCs, regardless of RAI status as determined by the ACTH stimulation test (19). Nevertheless, the diagnosis of adrenal insufficiency, the efficacy of GC therapy, and whether GC therapy should be stratified by adrenal insufficiency remain controversial.

The concept of RAI/CIRCI is vague (20), and the principal behind its diagnosis with the ACTH test is unclear (7, 21). It is essential to acknowledge that we may overlook a conceptual issue related to diagnosing RAI/CIRCI with the ACTH test. GCs are synthesized in the adrenal gland and are present in circulation at 20–200 ng/mL under physiological conditions. A striking feature of GCs is their inducible nature. In response to stress, adrenal glands produce high levels of GCs (induced GCs, iGCs) and catecholamines, a phenomenon that is collectively defined as stress response (22, 23). In our previous study, we specifically defined iGC production as an adrenal stress response in sepsis (24). The ACTH test measures the adrenal stress response (iGC production). While it is suitable for non-stressed patients, septic patients have endogenous iGCs due to septic stress; thus, the ACTH test measures an additional adrenal stress response on top of iGCs. What is the nature and function of “ΔiGC”? Does a low ΔiGC (<90 ng/mL) define “insufficient GC relative to an increased demand” or “inadequate cellular corticosteroid activity for the severity of the patient’s critical illness”?

Scavenger receptor BI (SR-BI or Scarb1) is the high-density lipoprotein (HDL) receptor, highly expressed in the liver and adrenal gland (25). It mediates the uptake of cholesteryl ester from HDL, which provides cholesterol for GC synthesis in the adrenal gland (26, 27). SR-BI whole body–null or adrenal gland–specific SR-BI–null mice have physiologic levels of corticosterone, but fail to produce iGCs in response to ACTH (24, 28–30), cecal ligation and puncture (CLP) (24, 29–31), or fasting (32). Thus, SR-BI is required for iGC production and SR-BI–null mice are an RAI or iGC insufficiency model. Using SR-BI–null mice, we demonstrated that iGC production is an essential host response that functions to control inflammatory response in sepsis (24, 29). Based on the mechanism of iGC production and function in sepsis, a normal adrenal stress response means producing sufficient iGCs to control inflammatory signaling. Conversely, RAI is defined by “insufficient iGC production to handle increased inflammation,” rather than by a low “ΔiGC.” We speculate that the ACTH test may misdiagnose adrenal insufficiency, potentially resulting in inappropriate use of GC therapy. If so, the inconclusive GC clinical trials might not be caused by targeting RAI/CIRCI, rather by a problematic ACTH test. To test this, we conducted the ACTH test in C57BL/6J mice and RAI mice. We found that the ACTH test fails to correctly diagnosis RAI at early/acute and middle stages of sepsis. Surprisingly, the ACTH test triggered lethal levels of inflammatory cytokine production in the adrenal gland and moderately increased mortality. Our findings suggest that the ACTH test may inaccurately diagnose adrenal insufficiency, leading to inappropriate use of GC therapy. The ACTH test–induced inflammatory cytokine production may further render the GC therapy ineffective in sepsis. Our study calls for developing a new method to diagnose adrenal insufficiency and to reevaluate GC therapy in sepsis.

## Results

### The ACTH test fails to correctly identify adrenal insufficiency in early/acute and middle/subacute stages of sepsis.

**Figure 1:**
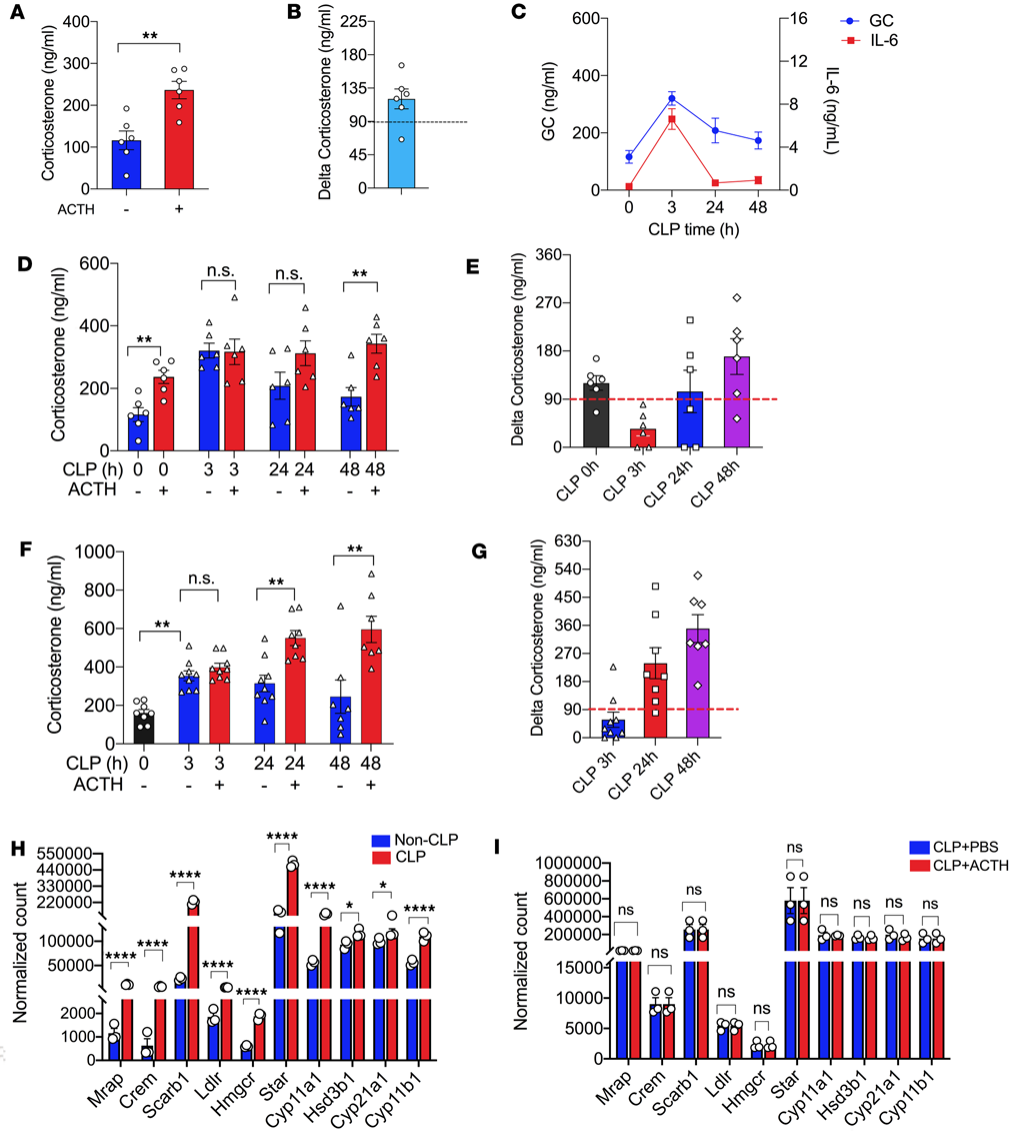
ACTH test fails to correctly identify adrenal stress response in early and middle stages of sepsis. (**A** and **B**) C57BL/6J mice were treated with 0.1 IU ACTH via subcutaneous injection. Serum corticosterone (**A**) and Δcorticosterone (**B**) levels were measured before and 1 hour after the ACTH test (*n* = 6). Data are presented as mean ± SEM. Statistical testing using 2-tailed unpaired Student’s *t* test. (**C**) C57BL/6J mice were challenged with CLP (25G, full ligation) for different times (0, 3, 24, 48 hours). Serum corticosterone and IL-6 levels were measured (*n* = 6–9). (**D** and **E**) C57BL/6J mice were challenged with CLP (25G, full ligation) for different times (0, 3, 24, 48 hours). Then, the mice were treated with 0.1 IU ACTH. Serum corticosterone (**D**) and Δcorticosterone (**E**) levels were measured before and 1 hour after the ACTH test (*n* = 6). (**F** and **G**) C57BL/6J mice were challenged with CLP for different times (0, 3, 24, 48 hours). Then, mice were treated with 4 IU ACTH. Serum corticosterone (**F**) and Δcorticosterone (**G**) levels were measured before and 1 hour after the ACTH test (*n* = 7–9). Data are presented as mean ± SEM. Statistical testing using 1-way ANOVA with Tukey’s multiple-comparison correction. (**H** and **I**) C57BL/6J mice were challenged with or without CLP for 3 hours (**H**) and stimulated with ACTH (4 IU) or PBS for 45 minutes (**I**). GC synthesis–related gene expression in the adrenal gland was analyzed by RNA-seq analysis. Data are presented as mean ± SEM. Statistics using 2-way ANOVA with Tukey’s multiple-comparison correction. NS, no significance; *P < 0.05; **P < 0.01; ****P < 0.0001. For RNA-seq analysis, pairwise comparisons between the various conditions were run using a negative binomial generalized log-linear model through the glmLRT fit function in edgeR (https://bioconductor.org/packages/release/bioc/html/edgeR.html).

First, we performed the ACTH test in wild-type (C57BL/6J) mice under physiological conditions. The mice exhibited a normal adrenal stress response to ACTH (0.1 IU) stimulation, as indicated by stress levels of corticosterone in circulation (Figure 1A) and a ΔGC of greater than 90 ng/mL (Figure 1B). Upon CLP challenge, sepsis induced stress levels of iGCs in circulation at the early/acute stage of sepsis (3 hours after CLP), with iGC levels gradually decreasing at 24 and 48 hours after CLP (Figure 1C). The mice displaced well-controlled inflammatory responses, as shown by high levels of interleukin-6 (IL-6) at the early/acute stage of sepsis (3 hours after CLP), with IL-6 levels under control at 24 and 48 hours after CLP (Figure 1C). Thus, the wild-type mice had a normal adrenal stress response and inflammatory response in sepsis. However, when we conducted the ACTH test in septic mice, the results were intriguing. At the early/acute stage of sepsis (3 hours after CLP), ACTH stimulation failed to induce additional GC production (Figure 1D), and all the septic mice had a ΔGC of less than 90 ng/mL (Figure 1E). Thus, all the septic mice were identified as having RAI or CIRCI by the ACTH test, even though the mice had a normal adrenal stress response to sepsis. At the middle/subacute stage of sepsis (24 hours aftert CLP), ACTH stimulation moderately increased GC levels (Figure 1D), but half of the mice were still identified as having RAI or CIRCI by the ACTH test (Figure 1E). At the late stage of sepsis (48 hours after CLP), ACTH stimulation induced a significant increase in corticosterone levels (Figure 1D), and most mice had a ΔGC of greater than 90 ng/mL (Figure 1E), thus being diagnosed with a normal adrenal stress response. It is worth noting that the wild-type mice had a normal adrenal stress response at all stages of sepsis, with no “insufficient GC relative to an increased demand” or “inadequate cellular corticosteroid activity for the severity of the patient’s critical illness.” Therefore, the ΔGC detected by the ACTH test is inappropriate for the diagnosis of adrenal insufficiency under septic conditions and may incorrectly diagnose RAI or CIRCI. Of note, the GC levels stimulated by ACTH (0.1 IU) were lower than those stimulated by CLP. Given that the clinical ACTH test dose is a super-stressor dose, we also performed the ACTH test with a super-stressor dose of ACTH (4 IU). The wild-type mice did not produce more iGCs at the acute stage of sepsis (3 hours after CLP) (Figure 1, F and G).

To understand why the ACTH test cannot stimulate more iGC production at the acute stage of sepsis, we looked at ACTH-mediated steroidogenesis in the adrenal gland. As shown in Figure 1H, upon CLP challenge, the expression of melanocortin 2 receptor accessory protein (*Mrap*) was upregulated by 10-fold, indicating the trafficking of the ACTH receptor (MC2R) to the cell membrane in response to septic stress. The downstream cAMP-responsive element modulator (*Crem*) was also upregulated by 10-fold. CLP induced 10-, 2.6-, and 3-fold increases in SR-BI (*Scarb1*, uptake of cholesterol from HDL), LDL receptor (*Ldlr*, uptake of cholesterol from LDL), and HMG-CoA reductase expression (*Hmgcr*, de novo cholesterol synthesis), respectively. CLP induced 2- to 3-fold increases in *Star* and *Cyp11b1* expression, the downstream key regulators of GC synthesis. Thus, sepsis induces robust ACTH-mediated steroidogenesis. However, all the above-listed key genes in GC synthesis were not further upregulated by the ACTH test (Figure 1I). Corticosterone is synthesized from cholesterol through a cascade of oxidative reactions that convert corticosterone precursors to corticosterone. These oxidative reactions are mediated by specific enzymes. We examined the expression of enzymes that convert corticosterone precursors to corticosterone. As shown in Figure 1I, the ACTH test did not induce more expression of the key enzymes, including *Cyp11a1* (catalyzes the conversion of cholesterol to pregnenolone, the first step in steroidogenesis), *Hsd3b1* (converts pregnenolone to progesterone), *Cyp21a1* (converts progesterone to 11-deoxycorticosterone), and *Cyp11b1* (converts 11-deoxycorticosterone to corticosterone, the final step in this pathway). This supports our conclusion that the ACTH test does not induce more activation of the hypothalamic-pituitary-adrenal (HPA) axis at the acute stage of sepsis.

We quantified ACTH levels at 3 and 24 hours after CLP (Supplemental Figure 2; supplemental material available online with this article; https://doi.org/10.1172/jci.insight.187487DS1). The sepsis-induced endogenous ACTH levels remained similar at both the acute and subacute stages of sepsis, consistent with previous reports in septic mice. This suggests that GC levels are regulated not only by endogenous ACTH levels but also by other factors. Early studies showed that IL-6, along with proinflammatory cytokines like TNF-α and IL-1β, stimulates the HPA axis, leading to increased production of GCs (33).

We noticed that the post-ACTH corticosterone levels seemed to remain rather at the same magnitude during the 48-hour follow-up in CLP animals, albeit different T0 levels. This suggests that the GC production may be capped by the stress levels. At the acute stage, the adrenal glands are in utmost stressed conditions so that the ACTH test could not induce more GC production. At 24 and 48 hours after CLP, the endogenous stress levels were significantly decreased, evidenced by low corticosterone concentrations and low inflammatory cytokine concentrations. At this point, the adrenal glands are ready to be stimulated again. Our earlier study showed that SR-BI–mediated uptake of cholesterol into the adrenal gland is a key step in the adrenal stress response (iGC production) in response to stress (30). To test our speculation, we examined adrenal SR-BI expression by Western blotting at 0, 4, and 20 hours after CLP. As expected, adrenal SR-BI expression was at high levels at 4 and 20 hours after CLP (Supplemental Figure 1). Upon stimulation by the ACTH test, iGCs returned to stress levels at 24 hours after CLP, and even higher at 48 hours after CLP by the super-stressor dose of ACTH at 4 IU (Figure 1, D–G).

We also noticed that the ACTH test stimulated higher GC levels in septic conditions than in physiological conditions (Figure 1, D and F). This suggests that the ACTH test acts together with other stimulators in sepsis to stimulate more GC production. As discussed above, IL-6, along with proinflammatory cytokines like TNF-α and IL-1β, stimulates the HPA axis, leading to increased production of GCs (33).

In sum, we demonstrated that the ACTH test fails to correctly identify adrenal insufficiency in early/acute and middle/subacute stages of sepsis.

### The ACTH test augments inflammatory cytokine production.

**Figure 2:**
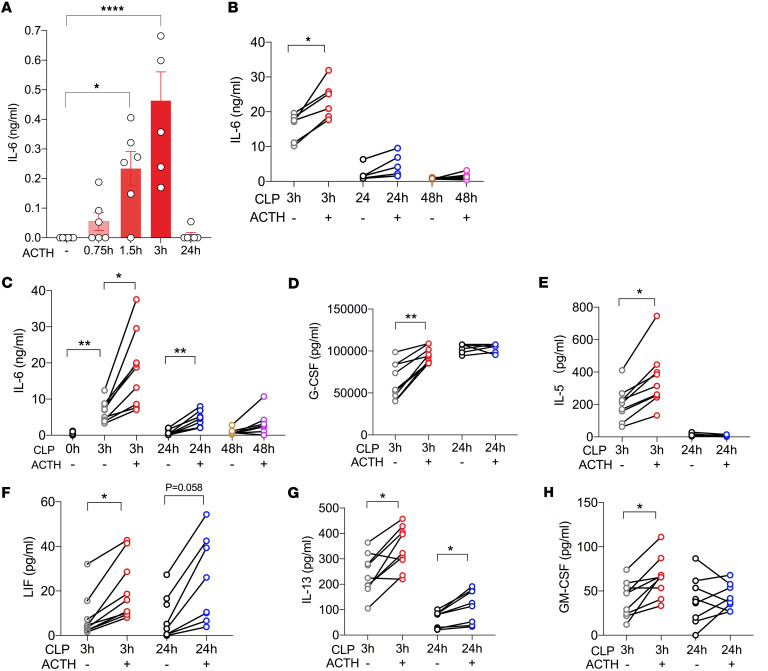
ACTH test augments inflammatory cytokine production. (**A**) C57BL/6J mice were treated with 0.1 IU ACTH. Serum IL-6 levels were quantified at the indicated times (*n* = 6). (**B**) C57BL/6J mice were challenged with CLP for 3, 24, and 48 hours and treated with 0.1 IU ACTH (*n* = 6). Serum IL-6 levels were quantified 1 hour later. (**C**–**H**) C57BL/6J mice were challenged with CLP for 3 and 24 hours and treated with 4 IU ACTH (*n* = 7–9). Serum cytokines were quantified 1 hour later by the 31-plex cytokine panel method. *P < 0.05; **P < 0.01; ****P < 0.0001 by 1-way ANOVA with Tukey’s multiple-comparison correction.

Unexpectedly, we found that the ACTH test rapidly and significantly increased IL-6 levels in circulation. Under physiological conditions, the ACTH test increased IL-6 levels to 0.2 ng/mL and 0.5 ng/mL at 1.5 and 3 hours after ACTH stimulation, respectively (Figure 2A). Under septic conditions, the ACTH test increased IL-6 to lethal levels, from 15.7 ng/mL to 23.4 ng/mL at 3 hours after CLP treatment (Figure 2B). We also investigated the effect of ACTH at a higher dose (4 IU) on cytokine production during the early stage of sepsis. Three or 24 hours after CLP, ACTH triggered more significant increases in multiple inflammatory cytokines, including IL-6 (Figure 2, C–H).

Together, we demonstrated that the ACTH test induces a significant increase in inflammatory cytokine production, to lethal levels under septic conditions.

### The ACTH test augments inflammatory signaling in the adrenal gland through transcriptional regulation of AP-1.

**Figure 3:**
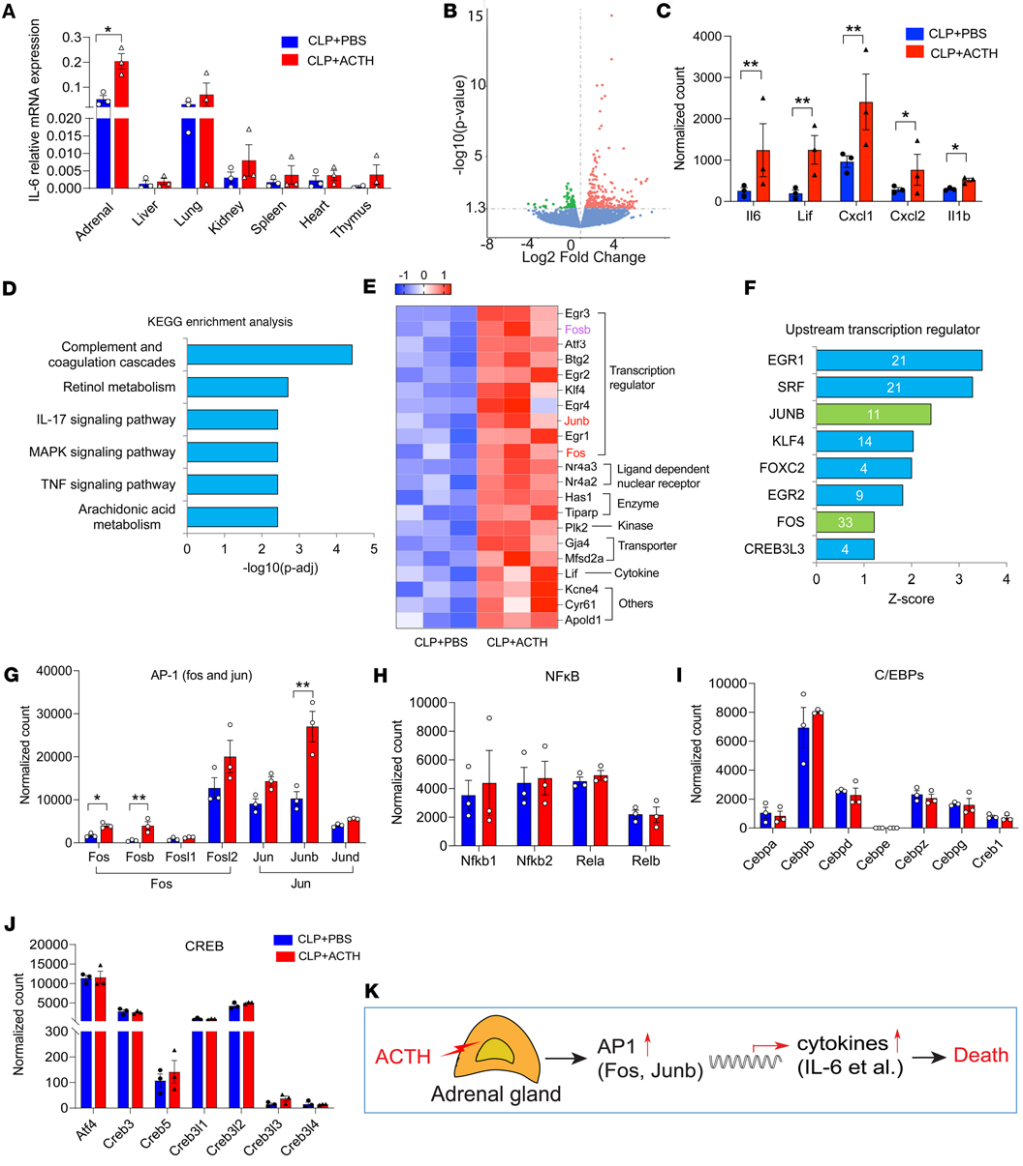
ACTH test augments inflammatory response in the adrenal gland through transcriptional regulation of AP-1 in sepsis. C57BL/6J mice were challenged with CLP (25G, full ligation) for 3 hours, and then treated with PBS or 4 IU ACTH. After 45 minutes, RNA from different tissues was isolated. (**A**) IL-6 mRNA expression was quantified by qRT-PCR. IL-6 relative mRNA expression was normalized to U36B4 expression and analyzed using a 2-tailed Student’s *t* test. (**B**–**J**) Adrenal RNA-seq analysis. (**B**) Volcano plot. (**C**) Differentially expressed cytokine genes. (**D**) The top 6 activated signaling pathways by KEGG enrichment analysis. (**E**) The top 21 differentially expressed genes in the adrenal gland. (**F**) The upstream transcriptional regulators analyzed by IPA. (**G**–**J**) Graphs showing upregulation of AP-1 signaling but not other signaling pathways (*n* = 3). *P < 0.05, **P < 0.01. RNA-seq analysis was performed using pairwise comparisons between conditions, applying a negative binomial generalized log-linear model with the glmLRT function in edgeR (https://bioconductor.org/packages/release/bioc/html/edgeR.html). (**K**) Schematic model of ACTH triggering inflammatory cytokine production in the adrenal gland.

We next investigated the mechanisms underlying ACTH promoting cytokine production. We first searched for the organ that produces IL-6 by quantifying IL-6 mRNA expression in different organs. Unexpectedly, among tested organs, we only observed a significant increase in IL-6 mRNA expression in the adrenal gland upon ACTH stimulation (Figure 3A). We then performed RNA-seq analysis to further investigate how ACTH stimulates inflammatory signaling. Compared with CLP mice, ACTH-treated CLP mice displayed 424 significantly differentially expressed genes, with 317 genes upregulated and 107 genes downregulated (Figure 3B). Among these, a number of inflammatory cytokines were significantly upregulated, including *Il6*, *Lif*, *Cxcl1*, *Cxcl2*, and *Il1b* (Figure 3C). KEGG enrichment analysis revealed activation of multiple signaling pathways in the ACTH-treated group (Figure 3D). The activation of complement and coagulation cascades and inflammatory signaling pathways is expected to contribute to worse outcomes in ACTH-treated mice. We evaluated the top 21 differentially expressed genes in the ACTH-treated group compared with the PBS-treated group. We found a group of transcription regulators among the top 21 differential expressed genes (Figure 3E). We then utilized Ingenuity Pathway Analysis (IPA) to assess the upstream transcription regulators. We found the upregulation of several transcription regulators in the ACTH-treated group, including early growth response factor (EGR) (*Egr1*, *Egr2*, *Egr3*, and *Egr4*), activator protein-1 (AP-1) (*Fosb*, *Fos*, *Junb*, and *Atf3*), and Krüppel-like factor 4 (*Klf4*) (Figure 3F). Inflammatory genes, including *Il6*, have been shown to be regulated by several transcription factors such as NF-κB, AP-1, cAMP response element (CRE)–binding protein (CREB), and CCAAT-enhancer-binding proteins (C/EBPs) (34, 35). We looked at the expression of these transcription regulators and found a significant upregulation of AP-1 family members (*Fosb*, *Junb*, and *Fos*) (Figure 3G), but no significant changes in the expression of NF-κB, CREB, and C/EBPs families (Figure 3, H–J). An early in vitro study showed that ACTH increases IL-6 release from cultured rat adrenal cells (36) and another in vitro study showed that ACTH induces the gene expression of *Fos* and *Junb* in cultured bovine adrenal fasciculata cells (37). These studies support our conclusion that ACTH augments cytokine production in the adrenal gland through AP-1 signaling.

Taken together, our results demonstrated that the ACTH test augments inflammatory cytokine production in the adrenal gland through AP-1 signaling (Figure 3K).

### The ACTH test augments inflammatory cytokine production in septic mice with RAI.

**Figure 4:**
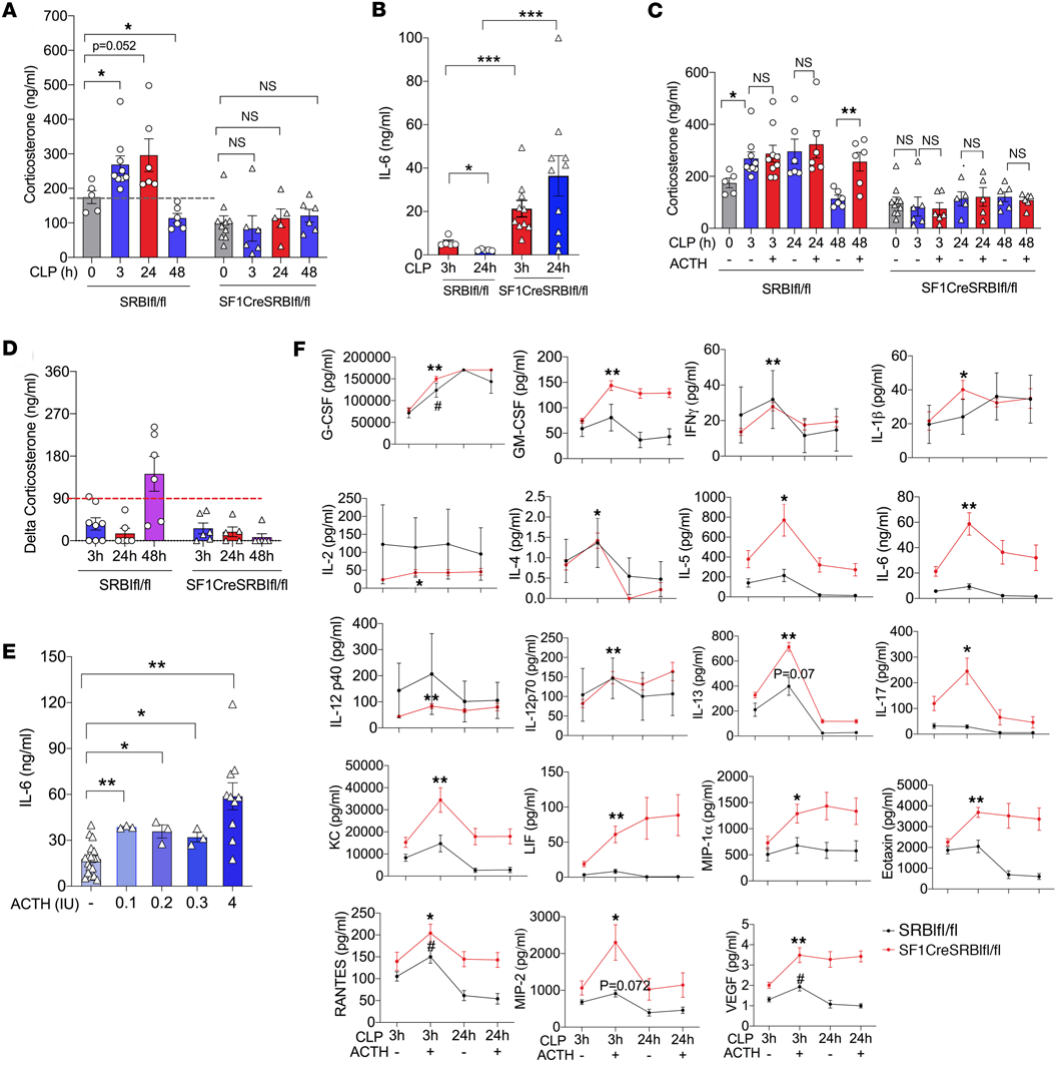
ACTH test augments inflammatory cytokine production in septic mice with RAI. (**A** and **B**) SRBI^fl/fl^ and SF1CreSRBI^fl/fl^ mice were challenged with CLP (25G, half ligation), serum corticosterone was measured at 3, 24, and 48 hours after CLP, and IL-6 levels were measured at 3 and 24 hours after CLP (*n* = 5–10). (**C** and **D**) SRBI^fl/fl^ and SF1CreSRBI^fl/fl^ mice were challenged with CLP (25G, half ligation); 3, 24, and 48 hours later, the mice were treated with 0.1 IU ACTH. Corticosterone was measured before and 1 hour after ACTH treatment (*n* = 5–10). (**E**) SF1CreSRBI^fl/fl^ mice (*n* = 3–19) were challenged with CLP for 3 hours and then treated with different doses of ACTH (0.1, 0.2, 0.3, 4 IU). Serum IL-6 levels were quantified 1 hour later. (**F**) SRBI^fl/fl^ (*n* = 5) and SF1CreSRBI^fl/fl^ mice (*n* = 10) were challenged with CLP (25G, half ligation); 3 and 24 hours later, the mice were treated with 4 IU ACTH and serum cytokines were measured 1 hour later with the 31-plex cytokine method. *P < 0.05; **P < 0.01; ***P < 0.001 by 1-way ANOVA with Tukey’s multiple-comparison correction (**A**–**F**). In **F**, *P < 0.05, **P < 0.01 for SF1CreSRBI^fl/fl^ mice; ^#^*P* < 0.05 for SRBI^fl/fl^ mice.

As an HDL receptor, SR-BI mediates the uptake of cholesterol from HDL, which is required for iGC synthesis under stress conditions (24, 26–32). Therefore, the adrenal gland–specific SR-BI–null (SF1CreSRBI^fl/fl^) mouse is a unique RAI model. Upon CLP challenge, wild-type (SRBI^fl/fl^) mice exhibited a normal adrenal stress response, indicated by stress levels of iGCs (Figure 4A) and well-controlled IL-6 (Figure 4B) in circulation. In contrast, SF1CreSRBI^fl/fl^ littermates showed adrenal insufficiency, with no iGC production (Figure 4A) and uncontrolled IL-6 (Figure 4B) in circulation. We then performed the ACTH test on CLP-challenged mice. At the early (CLP 3 hours) and middle stages (CLP 24 hours) of sepsis, almost all SRBI^fl/fl^ mice had a ΔGC of less than 90 ng/mL upon 0.1 IU of ACTH test (Figure 4, C and D). Even at CLP 48 hours, 1 out of 3 SRBI^fl/fl^ mice had a ΔGC of less than 90 ng/mL upon 0.1 IU of ACTH test. Thus, despite the wild-type mice having a normal adrenal stress response to sepsis and a well-controlled inflammatory response, they were incorrectly identified as RAI/CIRCI by the ACTH test at early, middle, and even late stages of sepsis. As expected, the ACTH test did not induce more iGC production in SF1CreSRBI^fl/fl^ under septic conditions (3, 24, and 48 hours after CLP) (Figure 4, C and D). Three hours after CLP, we treated the SF1CreSRBI^fl/fl^ mice with different doses of ACTH (0.1, 0.2, 0.3, and 4 IU). The ACTH treatment at 0.1 IU increased IL-6 to lethal levels, from 20 to 40 ng/mL, and the ACTH treatment at 4 IU increased IL-6 to 60 ng/mL in SF1CreSRBI^fl/fl^ mice (Figure 4E). In addition to IL-6, the 31-plex analysis showed that ACTH significantly increased 18 inflammatory cytokines in SF1CreSRBI^fl/fl^ mice (Figure 4F).

Taken together, these results demonstrated that ACTH test triggers more inflammatory cytokine production in septic mice with RAI.

### The ACTH test moderately decreases survival in septic mice.

**Figure 5:**
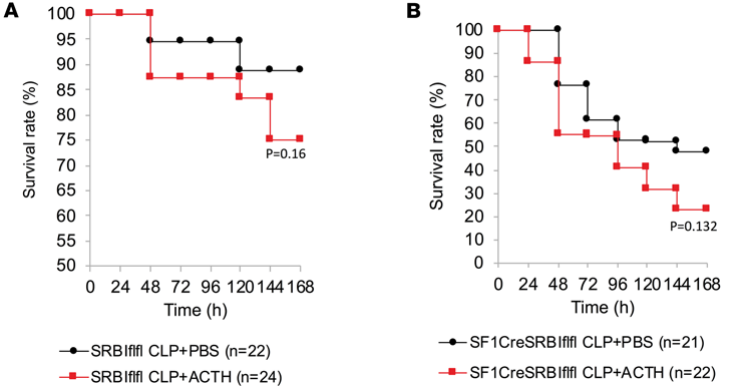
ACTH test moderately decreases survival in septic mice. SRBI^fl/fl^ (**A**) and SF1CreSRBI^fl/fl^ mice (**B**) were challenged with CLP for 3 hours (23G, half ligation in SRBI^fl/fl^ mice; 27G, one-third ligation in SF1CreSRBI^fl/fl^ mice). Then, mice were treated with PBS or 0.1 IU ACTH. Survival was monitored for 7 days and analyzed by log-rank test. The terms half ligation and one-third ligation refer to the points of ligation on the cecum based on the distance from its distal end.

Excessive production of inflammatory cytokines is associated with poorer outcomes in sepsis (38, 39). In this study, we assessed the impact of the ACTH test on the survival of SRBI^fl/fl^ and SF1CreSRBI^fl/fl^ mice. The ACTH test moderately reduced the survival rate from 89% to 75% in wild-type mice (Figure 5A) and from 48% to 23% in RAI mice (Figure 5B). We conclude that the ACTH test may have detrimental effects, as it significantly increases inflammatory cytokine production and moderately reduces survival in septic mice, regardless of RAI status.

## Discussion

### The ACTH test not only fails to correctly identify adrenal insufficiency but also triggers lethal levels of cytokine production in sepsis.

Early guidelines targeted RAI based on the French trial (8); however, the later CORTICUS trial did not confirm the survival benefit of GCs in septic patients with RAI (10). As a result, current guidelines no longer target RAI or CIRCI and instead recommend GC therapy based on the presence of shock. In this study, we questioned the nature of ΔiGC and speculated that the ACTH test is unsuitable for diagnosing RAI/CIRCI under septic conditions. We demonstrated that the ACTH test incorrectly identified most mice as having RAI/CIRCI during the early, middle, and even later stages of sepsis, despite these mice having a well-controlled inflammatory response and a normal adrenal stress response. Since GC therapy significantly reduced survival in septic mice with a normal adrenal stress response (24, 29), the ACTH test’s inability to correctly identify RAI could lead to misguided and potentially harmful GC use.

Importantly, we discovered that the ACTH test stimulates the production of inflammatory cytokines in the adrenal gland. Under septic conditions, the ACTH test elevated IL-6 to lethal levels, from 15.7 ng/mL to 23.4 ng/mL in wild-type mice, and from 20 ng/mL to 40 ng/mL in RAI mice. Excessive production of inflammatory cytokines is linked to poorer sepsis outcomes (38, 39). Indeed, ACTH treatment reduced the survival rate from 89% to 75% in wild-type septic mice and from 48% to 23% in RAI septic mice. Regarding the mechanism of how ACTH induces cytokine production, a previous in vitro study showed that ACTH increases IL-6 release from cultured rat adrenal cells (36) and another study showed that ACTH induces the expression of genes like *Fos* and *Junb* in cultured bovine adrenal fasciculata cells (37). Our study extends these in vitro findings by demonstrating that (a) the ACTH test induces multiple cytokines, including IL-6, through activation of the AP-1 and MAPK pathways in vivo; (b) the ACTH test induces lethal levels of IL-6 in the context of sepsis; and (c) the ACTH test moderately decreases the survival rate in septic mice.

While the dose of ACTH in the ACTH test is much higher than endogenous ACTH, endogenous ACTH may influence the systemic inflammatory response. Under septic conditions, endogenous ACTH may induce cytokines in the adrenal gland. Furthermore, endogenous ACTH is produced under non-septic conditions, such as psychological stress, which has long been associated with inflammation. Our findings may offer insights into the mechanisms behind stress-induced inflammatory responses. While ACTH is well known for its role in suppressing inflammation through the induction of GCs, understanding how endogenous ACTH augments inflammation may provide a novel target for managing inflammation in both sepsis and chronic diseases.

Taken together, our findings suggest that the inconclusive GC clinical trials may not be due to targeting RAI, but rather to a problematic ACTH test that (a) fails to correctly identify RAI, potentially leading to inappropriate use of GC therapy; and (b) promotes inflammatory cytokines to lethal levels, which renders the GC therapy less effective. Of note, the same ACTH test is used for the diagnosis of CIRCI (3, 7). Therefore, any problems with the ACTH test are also concerns for diagnosing CIRCI.

### There is an urgent need to develop a new diagnosis for adrenal insufficiency and to reevaluate GC therapy for patients with this condition.

Given the limitations of the ACTH test, we propose redefining adrenal insufficiency based on the mechanisms of GC generation and function. This approach classifies adrenal insufficiency into absolute adrenal insufficiency, iGC insufficiency, and GC resistance. Absolute adrenal insufficiency is characterized by a lack of physiological levels of GCs, as evidenced by patients with Addison disease, where physiological GC levels are essential for basal function and survival. This condition requires prompt GC therapy even under normal conditions (40). GC resistance, defined by impaired GC/GR signaling, currently lacks a diagnostic method, and GC therapy for these patients remains unexplored. Our focus is on iGC insufficiency. SR-BI–null mice, which have normal physiological GC levels but lack iGCs in response to stress, serve as a model for this condition (24, 28–32). We observed that both wild-type and iGC-insufficient mice exhibit strong inflammatory responses in the early stages of sepsis. While the inflammatory response diminishes in wild-type mice, it intensifies in iGC-insufficient mice during the middle stages of sepsis. This suggests that iGCs help control inflammatory responses and are produced in response to increased demand to counteract hyperinflammation in sepsis (24, 29, 41). Thus, we propose defining iGC insufficiency as insufficient iGC production accompanied by a hyperinflammatory response in sepsis. How do we diagnose iGC insufficiency? The iGC value is a random GC value under stress conditions. Although early efforts sought a cutoff GC value to define adrenal insufficiency (42), this is challenging, because the GC value changes with the process of sepsis, and more importantly, with the “demand” comes increased inflammation. Notably, CIRCI is characterized by a hyperinflammatory response due to a dysfunctional HPA axis. Although hyperinflammation is a key component of CIRCI, it is paradoxical that the diagnosis relies solely on random GC and ΔiGC values. We suggest using IL-6 as a marker for inflammation demand, combining a high IL-6 value with a lower-than-stress GC value to define iGC insufficiency. For example, in CLP-induced sepsis, an IL-6 value of greater than 3 ng/mL combined with a GC value of less than 200 ng/mL may indicate iGC insufficiency. Further studies are needed to validate this approach and determine cutoff values.

Given the dynamic nature of GC and cytokine production in sepsis, timely measurement of GCs and IL-6 is crucial. Technologies such as the FDA-approved Access IL-6 Assay, which measures IL-6 in 20 minutes, and a chip-based method that quantifies free cortisol in human saliva in minutes (43), offer practical solutions for bedside diagnostics.

In summary, given the issues with the ACTH test, there is an urgent need to develop a new diagnosis for adrenal insufficiency and to reevaluate GC therapy for patients. Since GC therapy benefits septic mice with iGC insufficiency but harms those without it (24, 29), we propose using precision medicine to guide GC therapy, specifically targeting patients with iGC insufficiency.

### Limitations.

The NIH has highlighted some limitations of using the mouse CLP model in sepsis studies. We believe that the mouse CLP model is suitable for studying the adrenal stress response in sepsis because: (a) Mice have physiological GC levels similar to those of humans (2–20 μg/mL); (b) iGC production is a key feature of sepsis in both humans and mice. Cortisol levels increase several-fold in septic patients (44) and corticosterone levels increase similarly in CLP-induced sepsis in mice (29, 30, 45), indicating a comparable adrenal stress response in both species. (c) Humans with a loss-of-function mutation in SR-BI exhibit reduced iGC production when stimulated by ACTH (46), indicating that SR-BI functions to regulate the adrenal stress response in both humans and mice. It is noteworthy that most wild-type (C57BL/6J) mice responded to ACTH stimulation at the late stage of CLP challenge due to diminished stress and upregulation of SR-BI expression. This may differ from septic patients, as some patients may still experience high stress at the late stage. We were unable to mimic these patients to evaluate whether ACTH augments inflammatory cytokine production at the late stage of sepsis using the current CLP model. Nonetheless, more preclinical and clinical research is needed to validate our findings.

## Methods

### Sex as a biological variable.

Both male and female animals and sex-matched littermates were used, if possible. Similar findings are reported for both sexes.

### Mice.

SF1-Cre mice were from The Jackson Laboratory (stock 012462). The mice were backcrossed for 6 generations against C57BL/6J mice from The Jackson Laboratory. SRBI^fl/fl^ mice were generated as described previously (47). The mice were backcrossed for 10 generations against C57BL/6J mice from The Jackson Laboratory. SF1-Cre mice were interbred with SRBI^fl/fl^ mice to yield SF1creSRBI^fl/fl^ and SRBI^fl/fl^ littermates. The animals were bred at the University of Kentucky’s animal facility. The animals were fed a standard laboratory diet and kept with a 10-hour/14-hour light/dark cycle and littermates were used at 10–14 weeks old.

### CLP sepsis model.

CLP was performed following a protocol described previously (31). No antibiotics were used for this study, because a significant number of septic patients have antibiotic resistance and, if antibiotics were used, more severe CLP conditions may be required that may increase the adrenal stress response. No postsurgery analgesia was used, as it interferes with the inflammatory response (48).

### ACTH stimulation test.

Mice were treated with or without CLP for different durations, followed by a single dose of ACTH administered via subcutaneous injection. Pilot experiments were conducted to determine the appropriate ACTH dose, referencing a previous study (28). It was found that ACTH (80–95 IU/mg; Sigma-Aldrich, A6303) at 0.1 IU induced GC to stress levels, but below those observed in CLP-induced sepsis. ACTH at 4 IU induced GC levels similar to those induced by CLP, indicating that 4 IU of ACTH is a super-stressor dose, comparable to the 250 μg dose used clinically. Therefore, ACTH at 0.1 IU was used as a stress level and at 4 IU as a super-stressor level in this study. Serum corticosterone was quantified using a Corticosterone ELISA kit (ENZO Life Sciences, ADI-900-097).

### Cytokine assay.

Serums were sent to Eve Technologies for cytokine analysis using the Mouse Cytokine Array/Chemokine Array 31-Plex method.

### RNA-seq analysis.

Six C57BL/6J mice in each group were challenged with CLP (25G needle, full ligation) for 3 hours, followed by ACTH (4 IU in 100 μL PBS) or 100 μL PBS treatment. After 45 minutes, total RNA was then isolated from adrenal glands with an RNeasy Mini Kit (QIAGEN, 74104). RNA samples were sent to Novogene for RNA-seq analysis. The data were also analyzed using Ingenuity Pathway Analysis (IPA) software (https://digitalinsights.qiagen.com/). Total RNA was also isolated from different organs and used for qRT-PCR analysis of *Il6* expression using *U36B4* as an internal control.

### Statistics.

Significance in experiments comparing 2 groups was determined by 2-tailed Student’s *t* test. Significance in experiments comparing more than 2 groups was evaluated by 1-way ANOVA, followed by post hoc analysis using Tukey’s test. Means were considered different at a *P* value of less than 0.05. Kaplan-Meier plots and log-rank tests were used to compare the survival curves. All statistical analyses were conducted on biological replicates using GraphPad Prism or SPSS software. All statistical tests were 2-sided with a significance level of 0.05.

### Study approval.

Animal care and experiments were approved by the Institutional Animal Care and Use Committee of the University of Kentucky, Lexington, Kentucky, USA.

### Data availability.

RNA-seq data generated in this study are deposited in the NCBI GEO public database (accession number: GSE290066). Values for all data points in graphs are reported in Supporting Data Values file.

## Author contributions

DH contributed to experimental design, conducting experiments, data analysis and interpretation, and manuscript writing. QW, MI, JX, and LG conducted experiments and analyzed data. CM and PWS generated SRBI^fl/fl^ mice. BH provided advice on statistical analysis. XAL contributed to study conception, experimental design, data interpretation, and manuscript writing.

## Supplementary Material

Supplemental data

Unedited blot and gel images

Supporting data values

## Version 1 03/06/2025

In-Press Preview

## Version 2 04/22/2025

Electronic publication
