# Supplementary material for: The ACTH test fails to diagnose adrenal insufficiency and augments cytokine production in sepsis
Source: JCI Insight. 2025 Mar 6;10(8):e187487. doi: 10.1172/jci.insight.187487 (PMC12016919; doi:10.1172/jci.insight.187487)
Supplement: Supplemental data [file jciinsight-10-187487-s038.pdf]

Supplemental Figures

Anti-SR-BI used in the western blot was customer made from Sigma. The antibody was verified with tissues from SR-BI null mice.

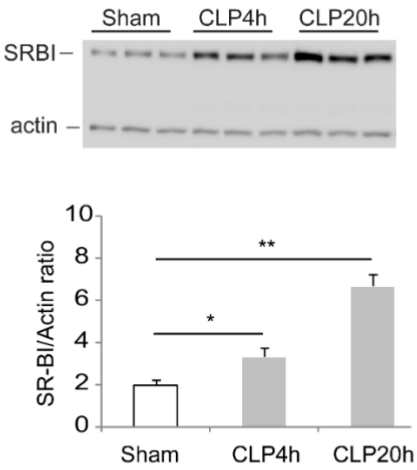

Supplemental Fig 1. C57BL/6J mice were challenged with cecal ligation and puncture (CLP) and the adrenal gland were harvested at the indicated times and subjected for western blot analysis. The expression of adrenal SR-BI was normalized by the expression of actin. (n=3). *p < 0.05, **p < 0.01

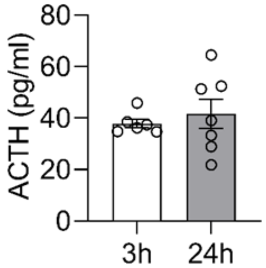

Supplemental Fig. 2. C57BL/6J mice were challenged with CLP for 3 and 24 hours and serum ACTH levels were quantified. Data are presented as means ± SEM.
