# Supplementary material for: The ACTH test fails to diagnose adrenal insufficiency and augments cytokine production in sepsis
Source: JCI Insight. 2025 Mar 6;10(8):e187487. doi: 10.1172/jci.insight.187487 (PMC12016919; doi:10.1172/jci.insight.187487)
Supplement: Unedited blot and gel images [file jciinsight-10-187487-s039.pdf]

#1 gel

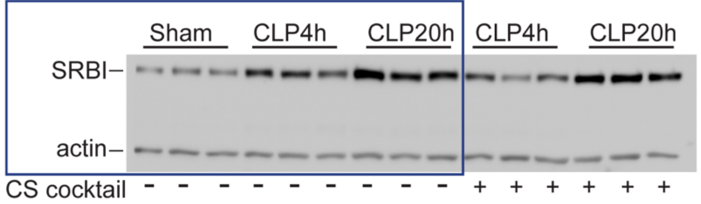

#1 full gel

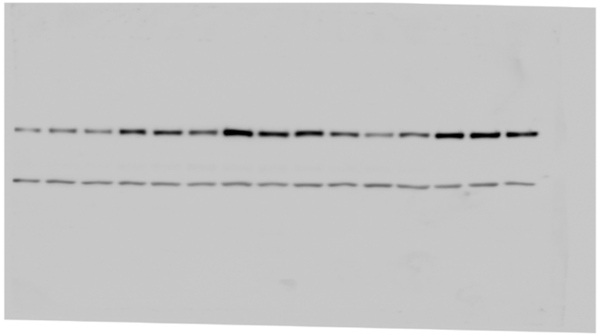

Original blot for Supplemental Fig 1. C57BL/6J mice were pre-treated with/without CS cocktail and challenged with CLP for indicated time. The adrenal gland were lysed and subjected to western blot analysis using anti-SR-BI antibody.
